# Comparison of immersive and non-immersive virtual reality videos as substitute for in-hospital teaching during coronavirus lockdown: a survey with graduate medical students in Germany

**DOI:** 10.1080/10872981.2022.2101417

**Published:** 2022-07-18

**Authors:** Albert J. Omlor, Leonie S. Schwärzel, Moritz Bewarder, Markus Casper, Ellen Damm, Guy Danziger, Felix Mahfoud, Katharina Rentz, Urban Sester, Robert Bals, Philipp M. Lepper

**Affiliations:** a Department of Internal Medicine V – Pneumology, Allergology and Intensive Care Medicine, Saarland University Medical Center, Homburg, Germany; b Department of Internal Medicine I - Hematology, Oncology, Clinical Immunology, Rheumatology, Saarland University Medical Center, Homburg, Germany; c Department of Internal Medicine II, Saarland University Medical Center, Homburg, Germany; d Department of Internal Medicine, Saarland University Medical Center, Homburg, Germany; e Department of Internal Medicine III - Cardiology, Angiology and Intensive Care Medicine, Saarland University Medical Center, Homburg, Germany; f Department of Internal Medicine IV - Nephrology and Hypertension, Saarland University Medical Center, Homburg, Germany

**Keywords:** Virtual reality, VR 180, Google Cardboard, Covid-19, medical education

## Abstract

As a consequence of the continued Covid-19 lockdown in Germany, in-hospital teaching for medical students was impossible. While lectures and other theoretical training were relatively easily converted into online sessions using platforms such as Moodle, Zoom and Microsoft Teams, this was not the case for practical skills and clinical interventions, such as bronchoscopy or colonoscopy. This study describes a workaround that was implemented at the Saarland University Hospital utilizing virtual reality equipment to convey the impressions of shadowing clinical procedures to the students without physical presence. To achieve this, 3D 180° videos of key clinical interventions of various internal medicine specialities were recorded, cut, and censored. The videos were uploaded to the e-learning YouTube channel of our institution and shared with the students via the private share function. The students could choose whether to use a VR-viewer to watch the videos immersively or to watch them without a viewer on a screen non-immersively. At the end of the course after 1 week, the students completed a questionnaire anonymously focusing on learning-success regarding the presented topics, a self-assessment, and an evaluation of the course. A total of 27 students watched the videos with a VR-Viewer and 74 watched non-immersively. Although the VR-viewer group self-assessed their expertise higher, there was no significant difference between the two groups in the learning-success test score. However, students in the VR-viewer group rated the learning atmosphere, comprehensibility, and overall recommendation of the course significantly higher. They also agreed significantly more to the statement, that they gained a better conception of the presented procedures, and that virtual reality might be an appropriate tool for online teaching. Video-assisted teaching facilitates learning and might be a valuable add-on to conventional teaching.

**Abbreviations:** Covid-19: severe acute respiratory syndrome coronavirus 2; 3D: three-dimensional; 2D: Two-dimensional; VR: virtual reality

## Background

Virtual reality was proposed to enhance medical education. The term Virtual Reality or VR is not uniquely defined. According to most sources, it refers to an interactive artificial experience of reality that induces a sense of presence [1]. Often, 360° technology and head mounted displays are used, so the viewer can change his perspective in a virtual scene within a complete 360° angle [2,3]. Those virtual reality scenes can be completely computer generated but also real environments recorded with special camera equipment. Virtual reality can be either immersive or non-immersive. Immersive VR is usually achieved with VR viewers that have a head mounted display. The viewer experiences a panoramic image as if inside the environment. When tilting the head, the virtual perspective adapts to this new position to create a strong illusion of presence [4]. The immersion can be even increased when a stereoscopic three-dimensional (3D) effect is created by displaying slightly different images to the left and right eye over the head mounted display. Alternatively, a recorded or simulated VR scene can also be played non-immersively on a standard display. Here, the viewing direction is changed manually via a mouse and/or keyboard on a computer or by rotating and tilting on smartphones or tablets. The viewer has the full perception of his real environment but not the impression of being in the virtual scene [4].

Recently, many viewing and recording devices for virtual reality have become commercially available. While the first virtual reality headsets required powerful hardware, newer developments work autonomously with all data processing integrated into the viewer. Modern smartphones come with all the necessary active components of a VR-viewer such as screen, processor, and sensors. Therefore, a working head mounted display can be created by adding lenses and a dedicated frame to a smartphone. Using this technique, Google Cardboard has been introduced in 2014, which at first, used a headset made of cardboard in combination with a smartphone [5].

**Figure 1.**
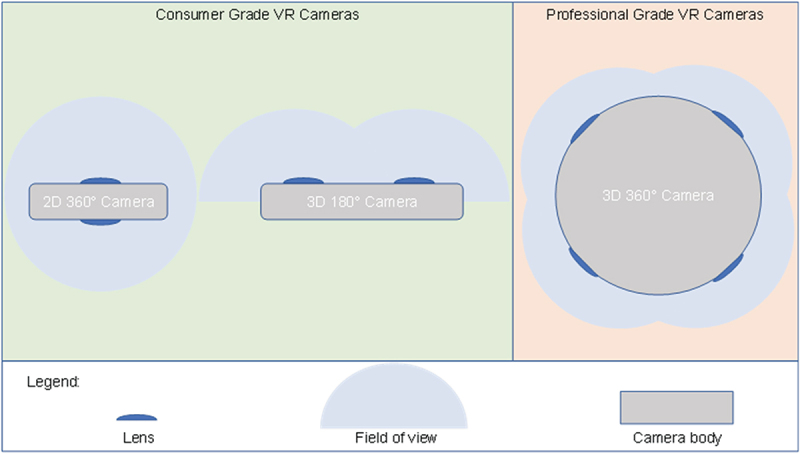
Overview of the available VR cameras. As consumer products both 2D 360° cameras and 3D 180° cameras are sold. 3D 360° cameras are sold for the professional market. For this project we chose a 3D 180° camera as the best feature price compromise.

In parallel to the development of VR viewers, cameras for VR also emerged. The most common type of those cameras uses two opposite wide angle camera sensors to capture a complete two-dimensional (2D) 360° panorama. The recording of 360° in stereoscopic 3D is technically complex and requires at least four camera sensors. Therefore, as a technical compromise stereoscopic cameras with 180° 3D were developed which sacrifice the additional field of view for the 3D effect. Those cameras have two wide angle camera sensors next to each other, with a similar distance as the human eyes [6–8] (Figure 1).

So far, three applications for immersive virtual reality in medical education have been described in the literature. VR has been used to visualize 3D simulations of organs for teaching in radiology and anatomy [9]. Another scenario includes interactive computer simulations of surgical, endoscopic, and emergency procedures [10–12]. Finally, pre-recorded 360° (or 180°) videos can be used in teaching applications where practical vision is essential [11]. Some studies even suggest that VR allows more effective teaching than conventional education [13–16]. As a new development, immersive virtual reality technology has been suggested a tool to mitigate the restrictions of Covid-19 on medical education such as the shortages of material and the requirement of social distancing [14]. The present study aimed at describing an educational project that was implemented at the Saarland University Hospital utilizing low-cost virtual reality equipment to provide insights into clinical procedures to the students without physical presence.

## Methods

### Students

Medical students at Saarland University have to complete a course rotating through all major clinical disciplines including internal medicine. Two weeks of this course are completed at the Department of Internal Medicine. In addition to the usual lectures and seminars, the course featured a practical module where the students essentially observed physicians and various clinical interventions from close by during those 2 weeks in small groups (max. 4 students). During that practical module an important part was the students’ observation of interventions such as bronchoscopy, colonoscopy, or coronary angiography. However, due to the pandemic situation related to Covid-19 in Germany, in-hospital teaching for medical students was almost completely shut down. Therefore, lectures and other theoretical training were converted into online sessions using platforms such as Zoom, Moodle, and Microsoft Teams. The aim of the presented work was to also enable the teaching of clinical interventions in this module by means of virtual reality, without the students being present in the clinic.

### Creation of the VR media

**Figure 2.**
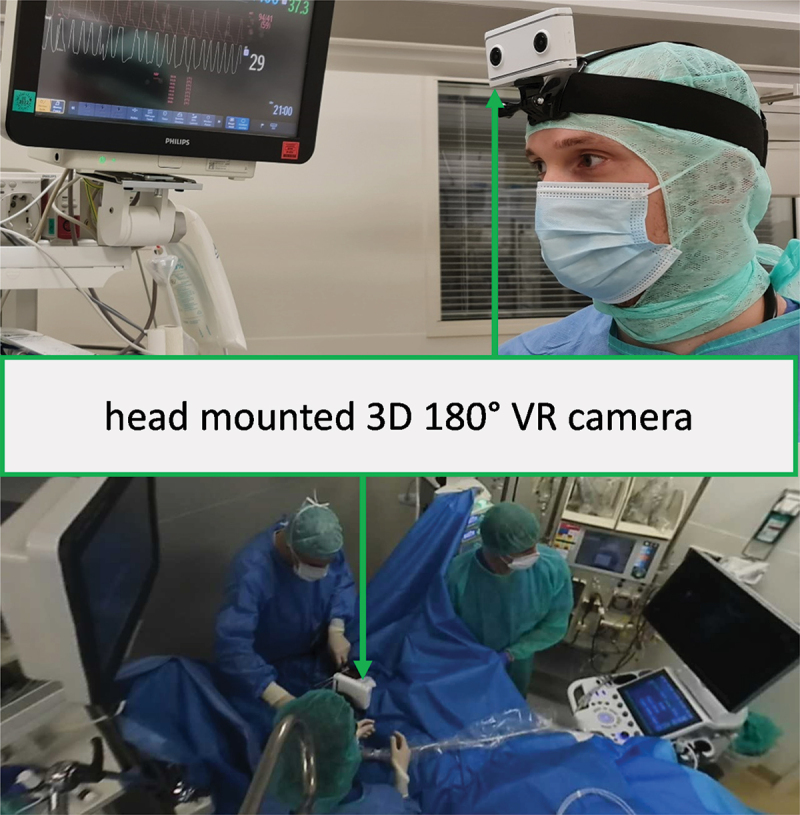
A 180° 3D camera worn on a head strap for video recording.

A 180° 3D camera, the Lenovo mirage camera (Figure 2), was used to create videos of key clinical interventions of all five departments of internal medicine. The camera was worn by either the physician during the intervention, or if this was not practical by a spectator close by. All patients and staff gave their written consent for the video recording. The topics of the videos are depicted in Table 1. All videos had to be post processed with the software Adobe Premiere Pro. This was not only necessary to shorten the videos, but also to add additional comments and most importantly to censor the faces of patients.

**Table 1.** List of videos created by the five departments of internal medicine.

Department	Type of intervention			
Haematology and Oncology	bone marrow puncture	bone marrow smear	microscopic examination of bone marrow	insertion of acentral venous catheter
Gastroenterology	EGD (endoscopic examination of oesophagus, stomach, and duodenum)	colonoscopy		
Cardiology	duplex sonography	echocardiography	cardioversion	
Nephrology	puncture of AV-fistula and haemodialysis	renal biopsy		
Pneumology	bronchoscopy	pleuracentesis	ECMO insertion	

**Figure 3.**
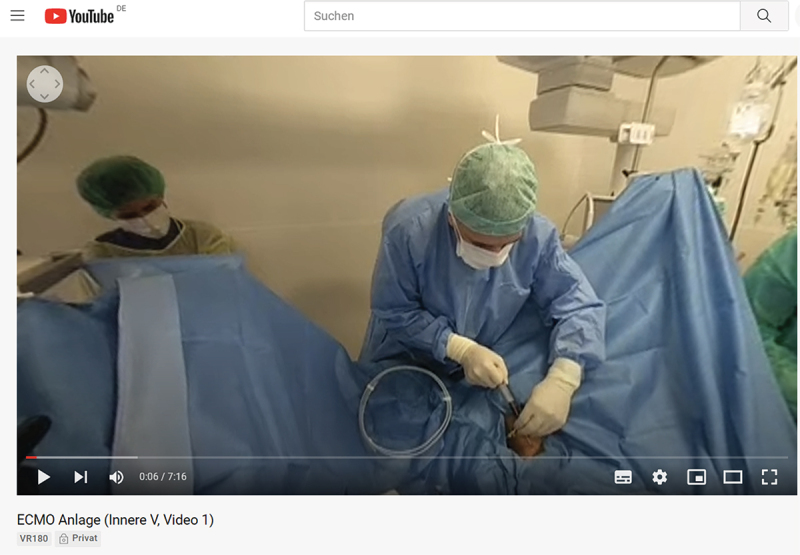
Screenshot of one of the recorded videos on the YouTube platform, viewed in 2D on a computer. The video was published as private, so only invited guests can watch it. The perspective can be changed by dragging the mouse or by clicking on the arrows in the top left part of the video.

With all patients’ and participants’ informed consent, the videos were uploaded to YouTube after the patients’ faces were censored. The videos were uploaded as private videos, so that only invited viewers were able to play them. VR 180° Videos can be viewed in three ways with YouTube: 1) In non-immersive 2D on a computer (Figure 3). The perspective can be changed with a mouse or keyboard. 2) In non-immersive 2D on a smartphone. The perspective can be changed by tilting the phone. 3) In immersive 3D with a virtual reality headset (e.g., a smartphone in a Google Cardboard viewer). The perspective can be changed by moving the head while wearing the viewer.

As a result of this, videos with a strong immersive effect were generated, where the viewer can actively change the view perspective. The students decided, if they watched the videos in non-immersive 2D or as immersive 3D VR using a Google Cardboard headset. The videos were part of a course that took 2 weeks.

### Evaluation

At the end of the course, the students completed a questionnaire that included a multiple-choice learning-success-test about the presented topics, a self-assessment, and an evaluation of the course. In the learning-success-test with 10 items, skills and knowledge that were either mentioned or demonstrated in the videos, were tested. The questions in German and their English translation are shown in Supplement Table S1. A learning success-sum score (0–10) was calculated as the sum of all correctly answered questions. Therefore, 0 was the worst possible scoring result and 10 the best.

In addition, the participant’s opinion about the quality of the course and their self-assessment were tested in 10 further questions using a Likert scale. Depending on the question, a higher number either indicated a better grade or a stronger consent (Supplement Table S2). The survey was voluntary and anonymous. No personal data were collected. The data collection started in May 2020 and ended in July 2020.

### Statistics

Convenience sampling was used as sampling technique. Statistical analysis was performed with SPSS Version 26 (IBM Inc, Armonk, NY). The sum score as well as the 10 Likert scale questions were treated as continuous variables. As the Shapiro–Wilk test rejected a normal data distribution, the Wilcoxon rank sum test was used to compare the 3D VR with the 2D group. A two-sided p-value <0.05 was considered significant.

## Results

Of the 105 medical students, who passed the online course in 5 groups of about 20 participants, 102 participated in the study. The demographic features of the learners are shown in Table 2. Twenty-seven students watched the videos in immersive 3D with a VR headset, while the rest watched it in non-immersive 2D on either a smartphone or a PC. The students in both groups did not assess their technical affinity differently. However, the students in the immersive group self-assessed their medical knowledge higher. Nevertheless, there was no significant difference in the sum score of the learning success between students who watched the VR videos immersively in comparison to students who did not.

**Table 2.** Demographic features of the students in the course.

Number (percentage) of students who participated in the survey	102 (97%)
gender distribution	
*male students*	41 (39%)
*female students*	64 (61%)
current semester of the students	
*students in their 5th clinical semester*	38 (36%)
*students in their 6th clinical semester*	65 (62%)
*students in their 7th clinical semester and higher*	2 (2%)

The immersive VR group gave an overall better evaluation of the course. This teaching method was generally favourably graded by the students. While the definition of the learning aims, the quality of the teaching material, and the overall learning success was not assessed differently, students in the immersive VR group gave a significantly better rating for the clarity and learning atmosphere of the course. In addition, students who watched the videos immersively would rather recommend the course than those in the non-immersive group. Moreover, students in the immersive VR group would agree significantly more that the videos improved their impression of the presented procedures, and that virtual reality is the right tool for teaching practical knowledge during a pandemic (Table 3).

**Table 3.** Differences in the scores of the items for learning success, course evaluation, and self-assessment between students who watched the videos in an immersive way with a Google Cardboard Viewer and those who viewed non-immersively on a Computer, Tablet or Phone.

	Score: median (IQB)		
Item (range)	Non-immersive group N = 74	Immersive group N = 27	*p*-Value
Learning-Success-Score (0–10)	7 (3)	8 (3)	*p* = 0.430
How good was the definition of the learning aims? (0–5)	4 (1)	4 (1)	*p* = 0.077
How well was the course structured and comprehensive? (0–5)	4 (1)	4 (1)	***p*** = 0.008
How would you assess the quality of the teaching media? (0–5)	4 (2)	4 (1)	*p* = 0.312
How would you assess your own expertise in the topic? (0–5)	3 (1)	4 (2)	***p*** = 0.040
How was the learning atmosphere? (0–5)	4 (2)	4 (1)	***p*** = 0.031
How would you assess your learning success? (0–5)	4 (1)	4 (0)	*p* = 0.064
Would you recommend the course? (0–5)	4 (2)	4 (1)	***p*** = 0.014
Do you possess a strong technical affinity? (0–5)	3 (1)	4, (1)	*p* = 0.209
Is virtual reality suitable for teaching practical knowledge during a pandemic? (0–5)	4 (1)	5 (1)	***p*** = 0.000
Did the videos improve your notion of the presented procedures? (0–5)	4 (0)	5 (1)	***p*** = 0.000

## Discussion

This project aimed to allow some degree of teaching of practical medical procedures when physical presence was prohibited. While immersive virtual reality using the Google Cardboard and similar platforms has been used in medical teaching before, this is the first study that describes the utilization of a low-cost immersive VR solution during the Covid-19 pandemic with hospital bans for students in place. So far, there are only few studies investigating the value of virtual reality to circumvent those hospital bans. De Ponti et al. described a non-immersive computer simulation. In this study, the clinical software Body InteractTM was used to allow pre-graduation students to play simulated clinical scenarios on normal displays [17]. Such a simulation used on ordinary computer screens does not feature the increased immersion achieved by head mounted displays. Another work used a proprietary immersive computer simulation for conducting a nasopharyngeal swab with Oculus Rift S head mounted device and hand controllers (Facebook Inc) [14]. A further study used Oculus Quest VR viewers to practice donning and doffing of personal protective equipment [18]. In our work, we also wanted to implement an immersive VR application. Due to limited resources and cost restrictions, an affordable and simple platform had to be identified.

**Figure 4.**
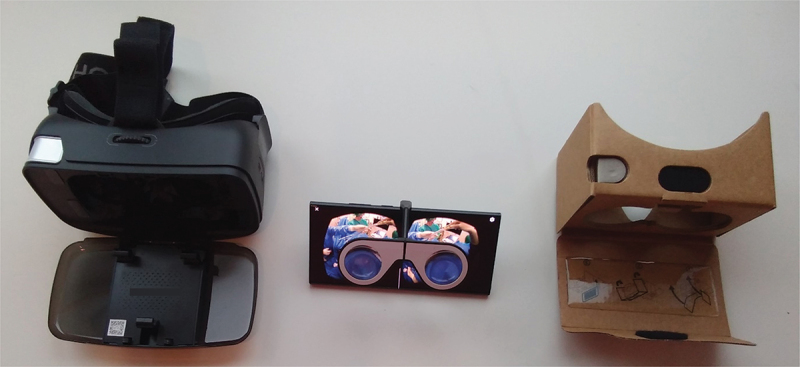
Three different Google Cardboard viewers: (from left to right) Homido V2, Homido mini, and Google Cardboard 2015.

We utilized the open-source Google Cardboard platform. This VR software can be used on smartphones with common operating systems (Android and IOS) in combination with cheap lens optics. Those viewers are available in several versions, with the cheapest ones made of cardboard can be obtained for under 10 € (Figure 4).

Moreover, the YouTube video platform supports Google Cardboard viewers. It was also clear during the development of this project that complex virtual reality setups that required programming of an application were not feasible. Hence, the idea was to capture a clinical scene in a 180° 3D video. In this case, the interaction of the viewers with the video is limited to changing the viewing direction providing some kind of interaction. The viewer must actively decide in which corner of the recorded 180° set the action takes place. This kind of interaction is sometimes called semi-immersive VR [19]. In contrast, in a conventional video recorded with a narrow angle lens, the camera operator would have to make this choice while the viewer has only a passive role [7]. Another important aspect of the VR 3D videos that we created was the stereoscopic 3D effect. Despite the limited resolution of the used VR viewer headsets, this 3D effect was very noticeable in all videos. Of the three common forms of VR cameras (2D 360° cameras, 3D 180° cameras, and 3D 360° cameras) only the latter two support stereographic 3D (Figure 1). However, without the 3D effect, we found the immersion of a 2D 360° camera too weak for this project. On the other hand, 360° 3D recording at the time was not available in consumer grade cameras but only in very expensive professional equipment. In the end, the 3D 180° camera Lenovo Mirage Camera was chosen for the project as the best compromise between features and price. All videos created for the project were 3D 180°. However, although we recommended the use of a VR viewer for the best experience, the students were given the free choice whether to follow the recommendation or rather watch the videos in 2D without a VR viewer. In fact, approximately one-third of the students chose to obtain and use a Google cardboard viewer.

Another important technical aspect was the distribution of the videos. While the preferred platforms for online teaching at our facility were Moodle and Microsoft Teams, neither of those supported the playback of 3D 180° videos. Because of its wide adoption and the excellent support of 3D 180° videos, YouTube was chosen to stream the created videos. However, while all patients and staff agreed to the online streaming of the videos, it was requested to provide access only to students at the Saarland University, which is why we used the ‘private share’ function of YouTube. All students were individually invited to watch the videos with their institutional email address.

In addition to the technical feasibility, we aimed to address the question whether the presented virtual reality videos augment teaching in times of a lockdown and whether the additional immersion by using VR viewers was worth the additional effort. While most students considered the VR videos as an appropriate medium for the course, the question regarding the add-on teaching value by using a VR-viewer headset was less clear. Students, who used a VR-viewer, evaluated both the quality of the media and the whole course better. Similarly, Birrenbach et al. documented in another study with immersive virtual reality, that the participants of the VR group were overall more satisfied with the course. In their study most of the students had no previous experience with VR. Therefore, the novelty effect, an increase in perceived usability due to the increased interest in a new technology, was discussed a possible explanation for the higher satisfaction [14]. The latter might also be a factor in our study. However, although we evaluated that the students herein did not receive any immersive virtual reality teaching in medical school prior to our study, we did not assess whether the participants used the technology for teaching in their previous schools or for entertainment at home. It should also be considered, that the Google cardboard viewers, as used in this study, are much more low-end and therefore potentially less impressive for the participants compared to the high-end Oculus Rift S devices in the other study.

Regarding the measurable teaching success in our study, the students in the VR-group achieved a numerically but not significantly higher score in the test part of the questionnaire. While interpreting the results, one has to keep in mind that the students were not randomized to an immersive VR-viewer or a non-immersive non-VR-viewer group.

Our study has potential limitations but although strength. A major limitation of our study is a selection bias of more self-motivated students being more likely to obtain a VR-viewer. Those students might also be more devoted to learning and therefore potentially have better scores overall. We tried to display this in the self-assessment part of the questionnaire. As expected, those students who decided to get a VR-viewer also assessed their own expertise higher than their counterparts. Therefore, this has to be considered when interpreting the study outcome.

Moreover, our analysis is not randomized and as a result observational and hypothesis generating. This causes imbalanced group sizes but, on the other hand, allow us to observe the willingness of students to choose VR as teaching modality.

In contrast to previous studies, it was not our aim to compare VR versus traditional teaching but to compare immersive and non-immersive VR. Therefore, there was no non-VR control group in our study.

## Conclusions

In conclusion, we present a viable and cost-effective method to provide immersive ‘almost bedside’ teaching to students via VR Videos in 180° 3D. While in this case it was used for a practical course in internal medicine, it could easily be adapted for medical education in any other discipline.

## Supplementary Material

Supplemental Material

## Data Availability

The datasets used and/or analysed during the current study are available from the corresponding author on reasonable request.
