# Supplementary material for: Comparison of immersive and non-immersive virtual reality videos as substitute for in-hospital teaching during coronavirus lockdown: a survey with graduate medical students in Germany
Source: Med Educ Online. 2022 Jul 18;27(1):2101417. doi: 10.1080/10872981.2022.2101417 (PMC9302008; doi:10.1080/10872981.2022.2101417)
Supplement: Supplemental Material [file ZMEO_A_2101417_SM4014.docx]

**Supplement Table 1**: Translated MC Questions for the Learning-Success-Test (and the original German Questions). The sum of all correct questions was calculated and called Learning-Success-Score.

| Question | MC answers (correct one bold) | | | | |
| --- | --- | --- | --- | --- | --- |
| How should the local anaesthesia for insertion of a central venous catheter be accomplished? *(Wie sollte die lokale Betäubung bei ZVK Anlage am ehesten erfolgen?)* | a)  Intravenous with 5mL Ultracain 2%  *(Intravenösmit 5mL Ultracain 2%)* | **b)**  **Subcutaneous with 5mL Ultracain 2%**  *(Subcutan mit 5 mL Ultracain 2 %)* | c) Subcutanous with 2.5 mL Ultracain 2% *(Subcutan mit 2,5 mg MSI)* | d)  Intravenous with 2.5 mg MSI *(Intravenös mit 2,5 mg MSI)* | e)  Topical with Voltaren Gel *(Topisch mit Volatren Gel)* |
| What is the anatomical leading structur for a bone marrow puncture? *(Was ist die anatomische Leitstruktur bei der Knochenmarpunktion am Beckenkamm?)* | **a)**  **posterior superior iliac spine**  *(Spina iliaca posterior superior)* | b)  posterior inferior iliac spine  *(Spina iliaca posterior inferior)* | c)  anterior inferior iliac spine  *(Spina iliaca anterior inferior)* | d)  anterior superior iliac spine  *(Spina iliaca anterior superior)* | e)  Sacrum  *(Os sacrum)* |
| What is the biggest hematologic cell in bone marrow? *(Was ist die größte hämatologische Zelle im Knochenmark?)* | a)  Macrophage  *(Makrophage)* | b)  Erythroblast  *(Erythroblast)* | c)  Promyelocyte  *(Promyelozyt)* | **d)**  **Megakaryocyte**  *(Megakaryozyt)* | e)  Plasma cell  *(Plasmazelle)* |
| Which gas is ideally used for insufflation in colonoscopy? *(Welches Gas wird optimalerweise zur Insufflation bei Koloskopie eingesetzt?)* | a)  O_2_ *(O_2_)* | b)  N_2_ *(N_2_)* | c)  Xe *(Xe)* | **d)**  **CO_2_ *(CO_2_)*** | e)  N_2_O *(N_2_O)* |
| Which statement about EGD and colonoscopy is NOT correct?  *(Welche Aussauge zu Koloskopie und ÖGD trifft NICHT zu?)* | a) The colonoscope can be bent in 2 planes by moving 2 wheels. *(Das Koloskop kann über 2 Rädchen in zwei Ebenen flektiert werden)* | **b)**  **The inspection in colsocopy is mainly done during forward movement.** *(Die Inspektion erfolgt bei der Koloskopie überwiegend beim Vorschieben.)* | c)  A loss of vascular drawing in colon is often the first sign of an inflammatory bowel disease.  *(Der Verlust der Gefäßzeichnung im Dickdarm ist meist das erste Zeichen einer entzündlichen Darmerkrankung.)* | d)  There are usually no gastric folds in the antrum. *(Im Antrum finden sich in der Regel keine Magenfalten.)* | e)  A teething ring is used to protect endoscope and teeth. *(Ein Beißring dient zum Schutz von Endoskop und Zähnen)* |
| Which statement about transesophageal echocardiography (TEE) and electrical cardioversion is NOT correct?  *(Welche Aussage zur transösophagealen Echokardiographie (TEE) und zur elektrischen Kardioversion trifft NICHT zu?)* | a) Due to increase droplet formation, safety precautions for Covid-19 have to be made. *(Da es zur vermehrten Tröpfchenbildung kommt müssen beim TEE Schutzvorkehrungen bzgl. Covid-19 getroffen werden.)* | b) In four-chamber view the atria are in the upper part of the image, hence close to the sonic head. *(Im TEE sind beim 4 Kammerblick die Vorhöfe im Bild oben, also schallkopfnah.)* | c) In order to show a right left shunt, such as a persistent foramen ovale, Gelafundin can be sterilely pushed between two syringes to create “bubbles” as contrast agent.  *(Zur Darstellung von rechts links Shunts, z.B. eines PFO, können durch steriles hin und her Drücken von Gelafundin in zwei Spritzen über einen Dreiwegehahn „Bubbles“ als Kontrastmittel erzeugt werden.)* | d) In electrical cardioversion conductivity can be optimised by additionally pushing the paddles on the adhesive electrodes. *(Bei der elektrischen Kardioversion kann die Leitfähigkeit verbessert werden, indem mit den Paddles zusätzlich auf die Klebeelektroden gedrückt wird.)* | **e) For electrical cardioversion, it is pivotal that the sync-function is always disabled on the defibrillator.** *(Es ist bei der elektrischen Kardioversion darauf zu achten, dass die Sync-Funktion am Defibrillator stets deaktiviert ist.)* |
| Which statement about the angiological examination of the neck arteries and veins is NOT correct? *(Welche Aussage zur angiologischen Untersuchung der Halsgefäße trifft NICHT zu?)* | a) The external carotid artery can by identified by a low negative diastolic flow and the existence of branches. *(Die Arteria carotis externa kann an einem niedrigen negativen Fluss in der Diastole und an dem Vorhandensein von Abgängen identifiziert werden.)* | **b) The subclavian artery usually has a biphasic flow profile.** *(Die Arteria subclavia zeigt normalerweise ein biphasisches Flussprofil.)* | c) The brain-supplying arteries, such as the interior carotid artery, have a high diastole. (Die Hirnversorgenden Gefäße, wie die Arteria carotis interna zeichnen sich durch eine hohe Diastole aus.) | d) The vertebral artery can be difficult to see due to running through the transverse foramina. *Die Arteria vertebralis kann durch ihren Verlauf durch die Foramina intervertebralia schwierig darzustellen sein.* | e) The common carotid artery shows a mixed profile between intenal and external carotid artery. *(Die Arteria carotis communis weist ein Mischprofil zwischen Arteria carotis interna und externa auf.)* |
| Which statement about the sonographic kidney biopsy is NOT correct?  *(Was trifft zur sonographisch gesteuerten Nierenbiopsie NICHT zu?)* | a) Both kidneys have to be sonographically examined. (Es müssen beide Nieren sonographisch dargestellt werden.) | **b) To avoid after-bleeding, patients have to remain in bed for 6 hours.** *(Um Nachblutungen zu vermeiden muss der Patient eine 6-stündige Bettruhe einhalten.)* | c) A few hours after the procedure a urine test, a blood count, and another duplex sonographie are performed. *(Wenige Stunden nach der Untersuchung erfolgt eine Begutachtung von Urin sowie ein Blutbildkontrolle und eine erneute Kontrollsonographie.)* | d) A stab incision of the skin with a scalpel is done before the actual puncture. *(Vor der eigentlichen Punktion wird mit einem Skalpell eine Stichinzision der Haut durchgeführt.)* | e) An AV-Shunt of the kidney blood vessels is a possible complication and has to sonographically ruled out. *(Auch eine AV-Fistel der Nierengefäße ist als Komplikation möglich und muss sonographisch ausgeschlossen werden.)* |
| Which statement about ECMO placement is NOT correct?  *(Was trifft zur ECMO Anlage NICHT zu?)* | a) Bladder syrings are used for both flushing the cannulae and for connecting the blood tubing. *(Blasenspritzen kommen sowohl beim Durchspülen der Kanüle als auch beim Verbinden der Blutschläuche zum Einsatz.)* | b) For placing a dual-lumen (Avalon) VV-ECMO, both the jugular vein and the inferior vena cava have to be sonographically examined. *(Bei der VV-ECMO mit Doppellumenkanüle (Avalon-Kanüle) muss sowohl die V. jugularis als auch die V. cava inferior sonographisch dargestellt werden.)* | **c) ECMO Therapy requires at least two cannulae.** *(Für die ECMO Therapie werden mindestens zwei Kanülen benötigt.)* | d) An Avalon-ECMO can be used in patients who are awake. *(Eine Avalon-ECMO kann am wachen Patienten betrieben werden.)* | e) In VV-ECMO and especially Avalon-ECMO the function of the lung is extracorporeally replaced. *(Bei der VV-ECMO und speziell bei der Avalon-ECMO wird extrakorporal die Funktion der Lunge ersetzt.)* |
| Which statement about bronchoscopy and pleurocentesis is NOT correct? *(Welche Aussage zu Bronchoskopie und Pleurapunktion trifft NICHT zu?)* | a) The flat trachealis muscle of the horseshoe-shaped trachea can be used for orientation during bronchsocopy. *(Man kann sich bei der Bronchoskopie an der flachen Pars membranacea der hufeisenförmigen Trachea orientieren.)* | b) Under certain circumstances it can make sense not to sedate the patient for bronchoscopy. (Unter bestimmten Umständen kann es sinnvoll sein den Patienten für die Bronchoskopie nicht zu sedieren.) | **c) The lingual segements of the left lung are the homologous feature to the lower lobe of the right lung.** (Die Lingula-Segmente auf der linken Seite entsprechen dem Unterlappen auf der rechten Seite.) | d) A pleurocentesis can be done on a sitting patient dorsal at the scapular line. *(Eine Pleurapunktion kann beim sitzenden Patienten dorsal in der Medioscapularlinie erfolgen.)* | e) During pleurocentesis the needle will be inserted at the upper border of the rib into the pleural cavity. *(Bei der Pleurapunktion punktiert man an der Oberkante der Rippe in den Pleuaraum.)* |

**Supplement Table 2**: Translated questionnaire items and original *(German items)* about teaching quality and students’ self-assessment

|  | Likert Scale | | | | |
| --- | --- | --- | --- | --- | --- |
| How good was the definition of the learning aims?  *(Wie gut wurden die angegebenen Lernziele definiert?)* | 5 excellent *(sehr gut)* | 4 good *(gut)* | 3 OK (*OK*) | 2 less good *(weniger gut)* | 1 poor *(schlecht)* |
| How well was the course structured and comprehensive?  *(Inwieweit war die Veranstaltung verständlich und übersichtlich bearbeitet?)* |  |  |  |  |  |
| How would you assess the quality of the teaching media? *(Wie bewerten Sie die Qualität der Unterrichtsmaterialien und -medien?)* |  |  |  |  |  |
| How would you assess your own expertise in the topic? *(Wie schätzen Sie Ihre eigene fachliche Kompetenz ein?)* |  |  |  |  |  |
| How was the learning atmosphere? *(Wie empfanden Sie die entstandene Lern- und Arbeitsatmosphäre?)* |  |  |  |  |  |
| How would you assess your learning success? *(Wie bewerten Sie Ihren Lernerfolg durch diese Veranstaltung?)* |  |  |  |  |  |
| Would you recommend the course? *(Würden Sie die Veranstaltung weiterempfehlen?)* | 5 absolutely *(auf jeden Fall)* | 4 mostly *(weitgehend)* | 3 in part *(Teile daraus sind eventuell wiederverwendbar)* | 2 rather not *(weniger)* | 1 not at all *(gar nicht)* |
| Do you possess a strong technical affinity? *(Sie würden sich selbst als technikaffin bezeichnen.)* | 5 strongly agree *(stimme völlig zu)* | 4 agree (stimme zu) | 3 neutral *(stimme weder zu noch nicht zu)* | 2 disagree *(stimme nicht zu)* | 1 strongly disagree *(stimme überhaupt nicht zu)* |
| Is virtual reality suitablel for teaching practical knowledge during a pandemic? *(Virtual Realitity eignet sich zur Vermittlung von praktischen Lerninhalten in Zeiten einer Pandemie.)* |  |  |  |  |  |
| Did the videos improve your notion of the presented procedures? *(Durch die erläuterten VR Inhalte haben Sie eine bessere Vorstellung der vorgestellten Prozeduren erhalten.)* |  |  |  |  |  |
